# Risk and reward: extending stochastic glycaemic control intervals to reduce workload

**DOI:** 10.1186/s12938-020-00771-6

**Published:** 2020-04-29

**Authors:** Vincent Uyttendaele, Jennifer L. Knopp, Geoffrey M. Shaw, Thomas Desaive, J. Geoffrey Chase

**Affiliations:** 1 GIGA-In Silico Medicine, University of Liège, Allée Du 6 Août 19, Bât. B5a, 4000 Liège, Belgium; 2 Department of Mechanical Engineering, University of Canterbury, Private Bag 4800, Christchurch, New Zealand; 3 Dept of Intensive Care, Christchurch Hospital, Christchurch, New Zealand; 4 School of Medicine, University of Otago, Christchurch, New Zealand

**Keywords:** Glycaemic control, Hyperglycaemia, Blood glucose, Insulin therapy, Insulin sensitivity, Insulin resistance, Workload, Trade-off

## Abstract

**Background:**

STAR is a model-based, personalised, risk-based dosing approach for glycaemic control (GC) in critically ill patients. STAR provides safe, effective control to nearly all patients, using 1–3 hourly measurement and intervention intervals. However, the average 11–12 measurements per day required can be a clinical burden in many intensive care units. This study aims to significantly reduce workload by extending STAR 1–3 hourly intervals to 1 to 4-, 5-, and 6-hourly intervals, and evaluate the impact of these longer intervals on GC safety and efficacy, using validated in silico virtual patients and trials methods. A Standard STAR approach was used which allowed more hyperglycaemia over extended intervals, and a STAR Upper Limit Controlled approach limited nutrition to mitigate hyperglycaemia over longer intervention intervals.

**Results:**

Extending STAR from 1–3 hourly to 1–6 hourly provided high safety and efficacy for nearly all patients in both approaches. For STAR Standard, virtual trial results showed lower % blood glucose (BG) in the safe 4.4–8.0 mmol/L target band (from 83 to 80%) as treatment intervals increased. Longer intervals resulted in increased risks of hyper- (15% to 18% BG > 8.0 mmol/L) and hypo- (2.1% to 2.8% of patients with min. BG < 2.2 mmol/L) glycaemia. These results were achieved with slightly reduced insulin (3.2 [2.0 5.0] to 2.5 [1.5 3.0] U/h) and nutrition (100 [85 100] to 90 [75 100] % goal feed) rates, but most importantly, with significantly reduced workload (12 to 8 measurements per day). The STAR Upper Limit Controlled approach mitigated hyperglycaemia and had lower insulin and significantly lower nutrition administration rates.

**Conclusions:**

The modest increased risk of hyper- and hypo-glycaemia, and the reduction in nutrition delivery associated with longer treatment intervals represent a significant risk and reward trade-off in GC. However, STAR still provided highly safe, effective control for nearly all patients regardless of treatment intervals and approach, showing this unique risk-based dosing approach, modulating both insulin and nutrition, to be robust in its design. Clinical pilot trials using STAR with different measurement timeframes should be undertaken to confirm these results clinically.

## Background

Critically ill patients often experience stress-induced hyperglycaemia [1]. Increased insulin resistance, antagonised insulin secretion, and excessive or unsuppressed hepatic glucose production all contribute to abnormally increase blood glucose (BG) levels. Hyperglycaemia is associated with increased morbidity and mortality [2, 3]. In 2001, glycaemic control (GC) demonstrated improved outcomes for these patients [4]. However, other studies failed to replicate the results [5–9], primarily blaming the increased risk of hypoglycaemia and glycaemic variability, both associated with worse outcomes [10–12]. These confounding outcomes have resulted in ongoing debate on GC [13–15], where current guidelines suggest higher glycaemic target bands and permissive hyperglycaemia due to fear of hypoglycaemia [16, 17].

A recent analysis suggests GC to lower glycaemic ranges was wrongly blamed for increased hypoglycaemia [18]. In this analysis, poor protocol compliance was pointed to as the most likely cause of hypoglycaemia. Hence, the association between increased hypoglycaemia and GC to lower glycaemic ranges in many randomised clinical trials could be biased by poor implementation. Another study showed overall GC outcomes do not rely on underlying patient condition, so critically ill patients who survive are not more or less difficult to control glycaemically than those who do not survive [19]. This implies that GC outcome, is a function of GC protocol design, not patient condition, indicating poor protocol design lacking personalisation as another culprit in poor study results, as well as that all patients should be able to benefit from well-designed (and implemented) control. More specifically, inter- and intra-patient variability is what makes GC hard to achieve safely [20, 21]. There is thus a critical need for personalised, *one method fits all*, glycaemic control [22]. Failing to provide safe, effective control for nearly all patients, regardless of which target band used, should thus not be acceptable, placing demand on better protocol design for safety and performance.

The Stochastic Targeted (STAR) GC framework is a model-based protocol directly accounting for both inter- and intra-patient variability [23]. STAR is a unique risk-based dosing approach, identifying patient-specific response to insulin, and forecasting likely future BG levels for specific insulin and nutrition inputs. STAR has been shown safe and effective for nearly all patients in three different countries and intensive care unit settings, despite targeting lower, normoglycaemic, ranges [24, 25].

To date, STAR uses 1–3 hourly forward prediction intervals to assess potential risk of hypo- and hyper-glycaemia for given 1–3 hourly treatments, averaging 11–12 BG measurements per day [23, 24]. While some ICUs can manage this workload, this value can be seen as excessive clinical burden for others, often due to lower nurse per patient ratios or greater clinical complexity of the patients. Equally, many clinical studies used longer intervals, but could not deliver safe, consistently effective GC [5–9].

This study extends from 1–3 hourly to 1–6 hourly measurement and intervention intervals in the STAR GC framework analyses the impact on GC safety and efficacy, using a clinically validated virtual patient modelling approach [26, 27]. If accomplished with minimally reduced safety and performance, this change has the potential to significantly reduce nurse workload, which is a major issue in GC [28–30]. It would also extend STAR’s capability while increasing its acceptability for clinical use in more ICUs. More specifically, this study aims to assess and quantify, for the first time, the risk (safety and performance) and reward (reduced workload) trade-off associated with lower BG measurement frequency, in the context of the original, proven, standard-of-care version of STAR [24].

## Results

### Stochastic models comparison

**Fig. 1:**
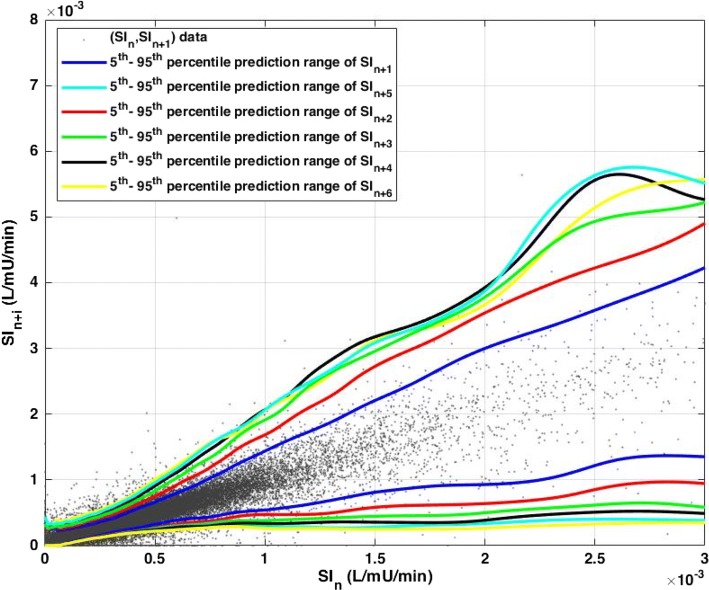
Stochastic model representation showing the 5th–95th percentiles prediction range of future 1–6 h SI levels given current identified patient-specific SI_*n*_

Stochastic models represent the probabilities of changes in insulin sensitivity (SI), as calculated from clinical data. Example 2D stochastic models for predictions 1–6 h ahead are presented in Fig. 1, where the 5th and 95th percentiles for future SI at a given current SI are shown. The probability distribution within these bounds would be described by a 3-D ‘mountain range’ sticking out of the page, approximately centred on the 1-1 line (as depicted in Fig. 8).

Intra-patient variability becomes more similar as prediction interval time increases, and the prediction lines converge to a similar range. This result clearly shows, while a bigger difference can be observed from 1 to 3 h in SI evolution, the difference in intra-patient variability becomes similar when longer intervals are considered. This outcome can represent a general, conservative, range of intra-patient variability, but alternatively may represent the average of more and less variable patients, which could result in reduced safety in some cases. More specifically, the longer interval model ranges may “hide” a larger range of changes (rising and falling) before returning to range, increasing the risk of larger unexpected glucose excursions.

A narrower range of possible SI outcomes translates directly to a narrower range of possible BG outcomes for a given treatment. As a result, more aggressive dosing can be used for shorter treatment intervals with narrower prediction of future SI variability ranges compared to longer intervals with wider prediction ranges. Thus, in general, the larger the measurement interval, the more conservative the treatment, given the likely higher potential sudden extreme changes in SI.

### STAR virtual trial results

**Fig. 2:**
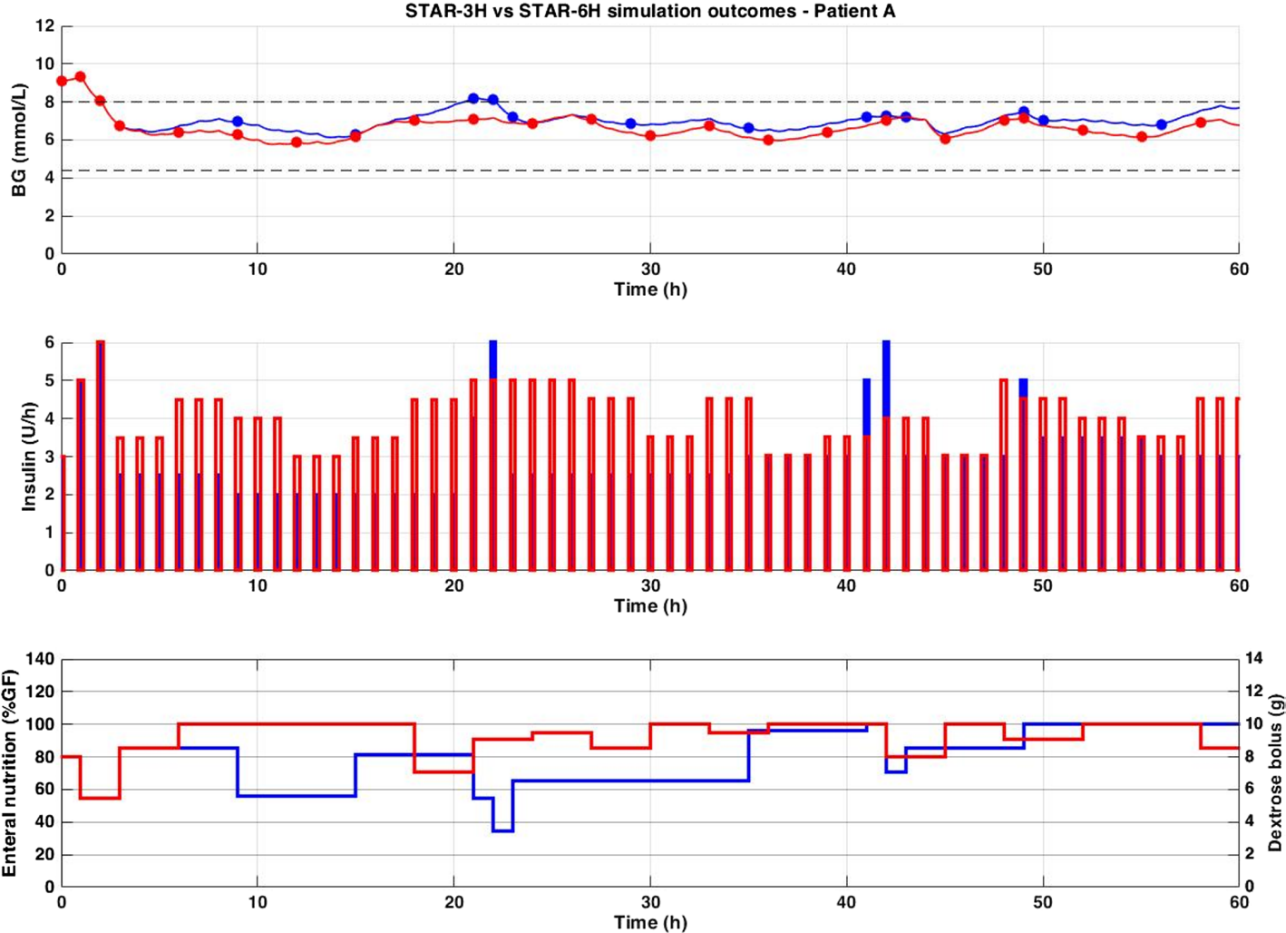
Excerpt of virtual trial results for Patient A. Blood glucose (top), insulin rates (middle), and enteral (solid line) and dextrose bolus (bars) nutrition rates (bottom) are compared between STAR-3H (red) and STAR-6H (blue)

**Fig. 3:**
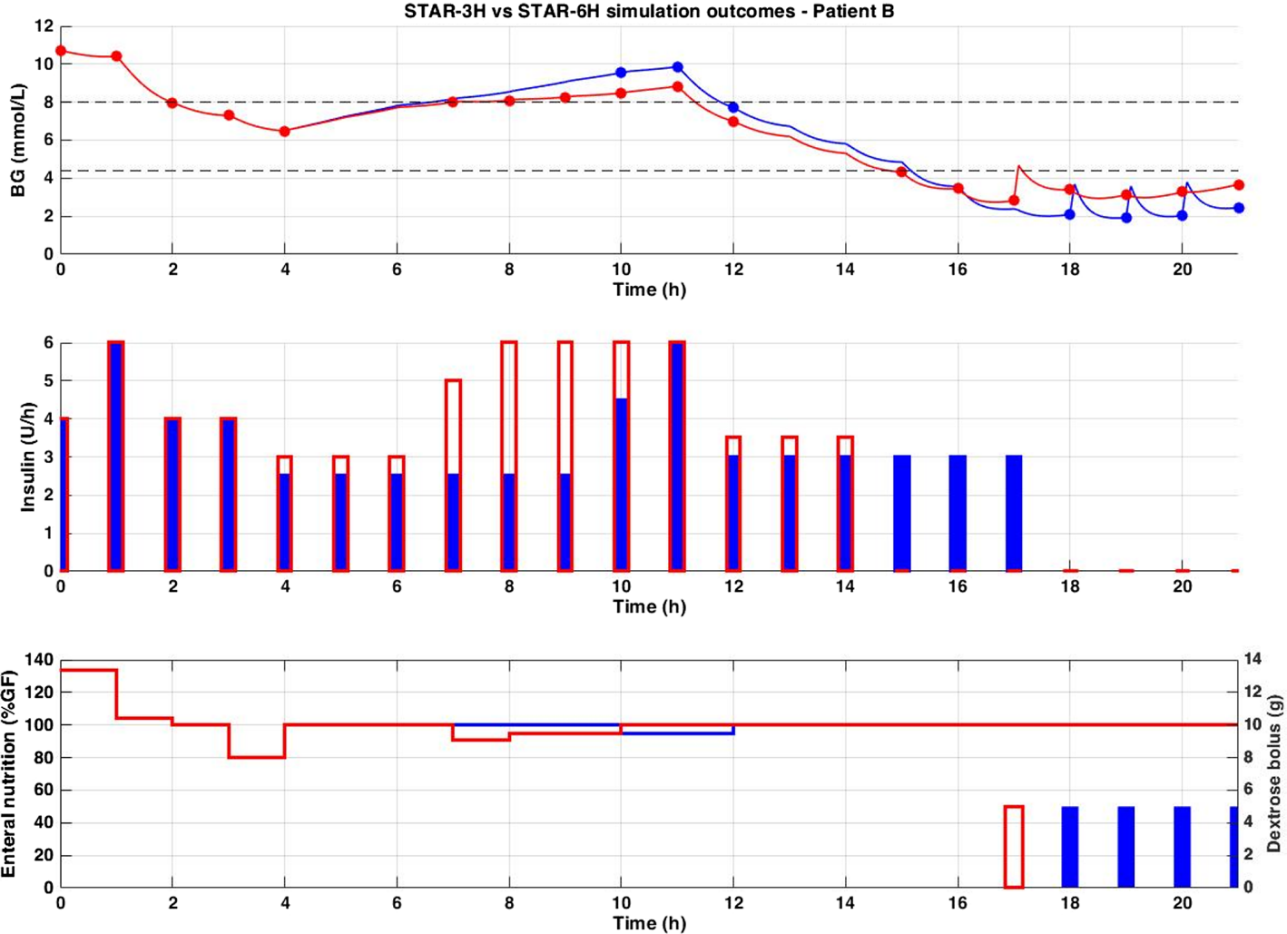
Excerpt of virtual trial results for Patient B. Blood glucose (top), insulin rates (middle), and enteral (solid line) and dextrose bolus (bars) nutrition rates (bottom) are compared between STAR-3H (red) and STAR-6H (blue)

Fivefold cross-validation virtual trial results using virtual patients, or ‘digital twins’ derived from clinical data, are presented in Table 1, for each version of STAR (1 to 3-, 4-, 5-, and 6-hourly). These digital twins allow analysis of BG response cohort to different treatment protocols to be compared in both individual patients and the overall cohort. Each arm has the same number of patients, but can have a slightly different number of GC hours, depending on the last measurement interval used in each virtual patient trial (i.e.: if last treatment is 3-hourly vs. 6-hourly, there will be 3 extra simulated hours of GC for this patient). Excerpts from two virtual patient trials comparing STAR-3H and STAR-6H are also presented in Figs. 2, 3.

**Table 1:** Virtual trial results of STAR Standard for 1 to 3-,4-,5-, and 6-hourly intervals

	STAR-3H	STAR-4H	STAR-5H	STAR-6H
# Episodes	681	681	681	681
# GC hours	59,240	59,528	59,782	60,003
# BG measures	28,961	24,792	22,243	20,272
Workload (meas. per day)	12	10	9	8
Median BG (mmol/L)	6.5 [5.9 7.3]	6.7 [6.1 7.5]	6.8 [6.2 7.6]	6.9 [6.3 7.7]
Median insulin (U/h)	3.2 [2.0 5.0]	3.0 [2.0 4.0]	2.5 [2.0 3.5]	2.5 [1.5 3.0]
Median nutrition (%GF)	100 [85 100]	95 [80 100]	90 [80 100]	90 [75 100]
%BG in 4.4–8.0 mmol/L	83	82	81	80
%BG in 4.4–7.0 mmol/L	65	59	55	52
%BG > 8.0 mmol/L	15	16	17	18
%BG < 4.4 mmol/L	1.6	1.5	1.5	1.6
%BG < 2.2 mmol/L	0.03	0.02	0.04	0.06
# Patients ≥ 50%BG in 4.4–7.0 mmol/L (%)	466 (68%)	432 (63%)	401 (59%)	372 (55%)
# Patients ≥ 50%BG in 4.4–8.0 mmol/L (%)	589 (86%)	583 (86%)	573 (84%)	571 (84%)
# Patients min BG < 2.2 mmol/L (%)	14 (2.1%)	12 (1.8%)	18 (2.6%)	19 (2.8%)

Results are based on hourly resampled BG. Median [IQR] is given for per-patient statistics, where appropriate

As expected, workload decreased as measurement interval increased (from 12 to 8 measurements per day for STAR-3H to STAR-6H). Time in the 4.4–8.0 mmol/L target band was high and similar in all scenarios (80–83%), but with a clear shift upward in median BG levels (6.5 [5.9 7.3] mmol/L for STAR-3H to 6.9 [6.3 7.7] mmol/L for STAR-6H), as reflected in the decreasing % BG in 4.4–7.0 mmol/L. Additionally, the number of patients with ≥ 50% BG in the tighter, safer 4.4–7.0 mmol/L (68% to 55%) and the wider, safe 4.4–8.0 mmol/L (86% vs 84%) slightly decreased, where additional analysis showed 80% of these patients dropping below 50% in those ranges were typically going to higher BG ranges, and 20% where going to lower BG ranges.

Incidence of hyperglycaemia is slightly higher as the interval increased. Most importantly, the incidence of severe hypoglycaemia increased as measurement interval increased, and the number of patients experiencing severe hypoglycaemia also increased (from 14 to 19 patients between STAR-3H and STAR-6H, 2.1% to 2.8% by patient). Interestingly, hypoglycaemia decreased in STAR-4H, with only 12 (1.8%) patients experiencing severe episode.

Overall, these results were achieved with lower insulin and nutrition rates as intervals increased. However, the nutrition rates remained high in these scenarios, where only 25% of patients received less than 75% patient goal feed (GF) in the worst case (STAR-6H). Thus, there was also some increased hyperglycaemia, as noted.

### STAR Upper Limit Controlled (STAR-ULC) virtual trial results

An ‘Upper Limit Controlled’ approach is also analysed, in which nutrition is modulated so the upper 95th percentile of possible BG outcomes does not exceed 8.5 mmol/L. This approach reduces hyperglycaemia, as well as the increased risk associated with large insulin and nutrition doses, which amplifies uncertainty in SI, especially as the measurement interval increases. Fivefold cross-validation results of the 1 to 3-, 4-, 5-, and 6-hourly versions of the STAR Upper Limit Controlled (STAR-ULC) approach, forcing the 95th percentile of BG ≤ 8.5 mmol/L are presented in Table 2.

**Table 2:** Virtual trial results of STAR-ULC 1 to 3-,4-,5-, and 6-hourly, forcing the predicted 95th BG percentile ≤ 8.5 mmol/L

	STAR-ULC-3H	STAR-ULC-4H	STAR-ULC-5H	STAR-ULC-6H
# Episodes	681	681	681	681
# GC hours	59,203	59,392	59,614	59,845
# BG measures	31,204	27,196	24,769	23,387
Workload (meas. per day)	13	11	10	9
Median BG (mmol/L)	6.4 [5.9 7.2]	6.5 [6.0 7.3]	6.5 [6.0 7.3]	6.5 [6.0 7.3]
Median insulin (U/h)	3.0 [2.0 4.5]	2.5 [1.7 4.0]	2.0 [1.5 3.5]	2.0 [1.5 3.5]
Median nutrition (%GF)	95 [80 100]	75 [65 85]	70 [60 80]	60 [50 75]
%BG in 4.4–8.0 mmol/L	84	84	85	85
%BG in 4.4–7.0 mmol/L	68	67	67	67
%BG > 8.0 mmol/L	14	14	14	14
%BG < 4.4 mmol/L	1.6	1.5	1.5	1.5
%BG < 2.2 mmol/L	0.02	0.02	0.02	0.04
# Patients ≥ 50%BG in 4.4–7.0 mmol/L (%)	497 (73%)	486 (71%)	481 (71%)	481 (71%)
# Patients ≥ 50%BG in 4.4–8.0 mmol/L (%)	597 (88%)	596 (88%)	594 (87%)	592 (87%)
# Patients min BG < 2.2 mmol/L (%)	11 (1.6%)	9 (1.3%)	9 (1.3%)	15 (2.2%)

Results are based on hourly resampled BG. Median [IQR] is given for per-patient statistics, where appropriate

High performance (~ 84% in target band and ~ 67% in 4.4–7.0 mmol/L) and high safety (14% BG > 8.0 mmol/L and 1.5% BG < 4.4 mmol/L) were achieved, and this result was surprisingly very similar regardless of measurement intervals. The number of patients experiencing severe hypoglycaemia decreased compared to STAR Standard (Table 1). STAR-ULC-4H (9 patients) and STAR-ULC-5H (9 patients) had both reduced number of patients experiencing hypoglycaemia compared to STAR-4H (12 patients) and STAR-5H (18 patients). These values were also lower compared to STAR-ULC-3H (11 patients) and STAR-ULC-6H (15 patients). This result reflects a reduction in risk due to reduced insulin dose by limiting the upper glycaemic as well within the STAR risk-based dosing system.

The number of patients with ≥ 50% BG in 4.4–7.0 mmol/L (~ 71%) and 4.4–8.0 mmol/L (~ 87%) was similar across all measurement intervals, reflecting effective control was achieved consistently for most patients. These numbers are higher compared to STAR Standard (Table 1), especially when comparing the tighter, safer 4.4–7.0 mmol/L band (55–68% for STAR Standard vs. 71–73% for STAR-ULC), which would reflect a significant improvement in outcomes [31, 32].

**Fig. 4:**
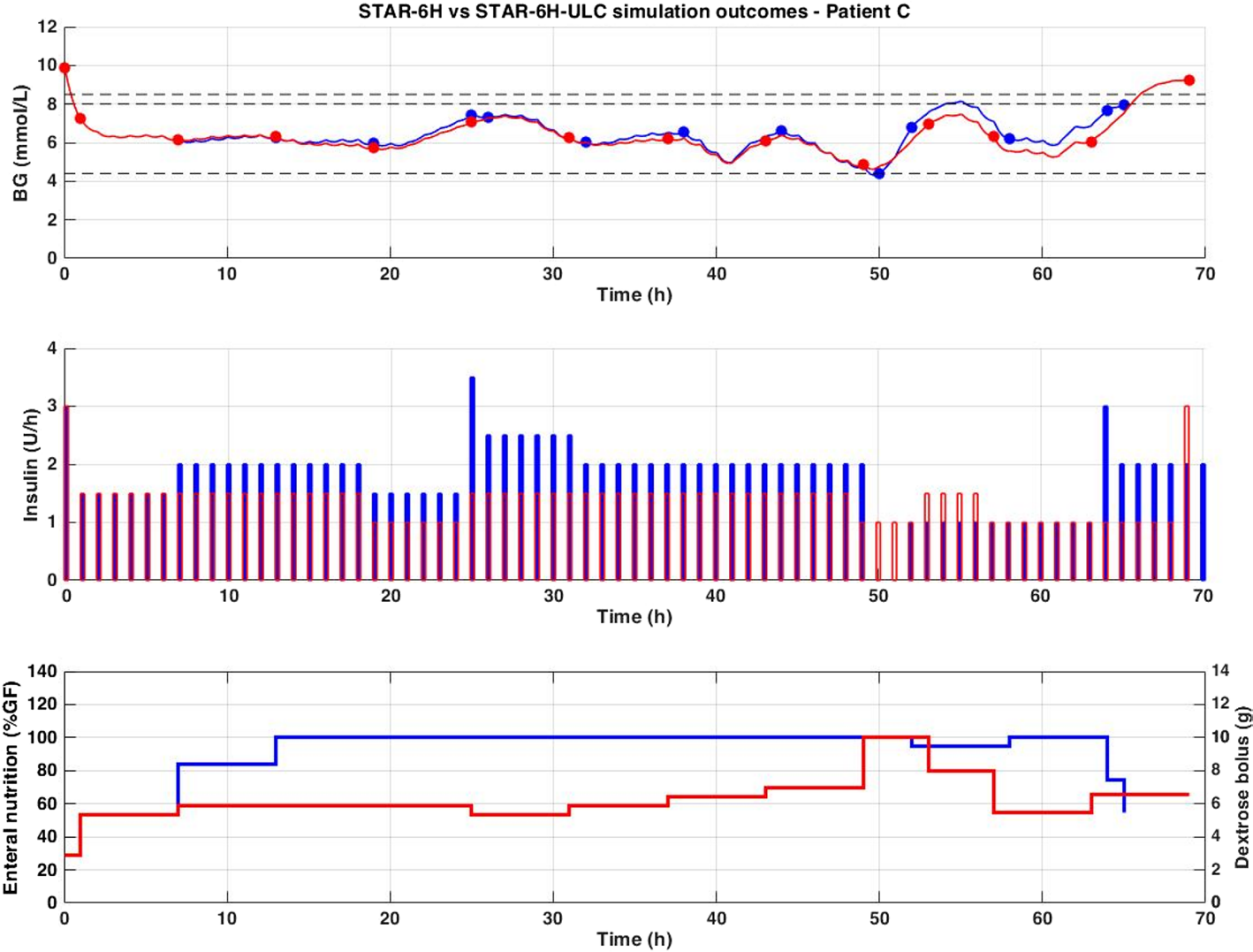
Excerpt of virtual trial results for Patient C. Blood glucose (top), insulin rates (middle), and enteral (solid line) and dextrose bolus (bars) nutrition rates (bottom) are compared between STAR-6H (blue) and STAR-ULC-6H (red). The 4.4–8.0 mmol/L target band is shown as well as the 8.5 mmol/L limit (dashed black)

Improved safety and efficacy were achieved here with significantly lower insulin and nutrition rates administered (Table 2) compared to STAR Standard (Table 1). A comparison of STAR-6H and STAR-ULC-6H is presented in Fig. 4, where this difference is clearly illustrated. Finally, workload increased by 1 additional measurement per day for each version compared to STAR Standard (Table 1), but are still lower than STAR 3-h standard of 12 per day [24] at the 4–6 hourly intervals with better performance and safety.

## Discussion

Foremost, it is important to understand metabolic variability, reflected in inter- and intra-patient variability, is what makes GC hard to achieve safely [19, 21]. Therefore, it is critical for GC protocol design to account for both, using dynamic, personalised solutions [22]. While the use of physiological models allows direct identification of inter-patient variability [20], STAR is the only current protocol [33] also using stochastic modelling to evaluate intra-patient variability [34, 35], which it then employs in a unique risk-based dosing strategy [23].

In a previous study comparing survivors and non-survivors, inter-patient variability has been shown different while intra-patient variability was clinically equivalent [19]. Therefore, this result emphasises the importance of identifying key physiological parameters, such as SI here, and assessing potential variability to provide safe, and effective control for all, which is critical to improving outcomes [18, 36]. In addition, compliance to protocol is essential to ensure any clinical judgement bias in results outcomes and conclusions [18], where longer intervals may improve compliance [37].

In many ICUs, protocols are often 4-hourly based once BG levels are stabilised in the target band. In practice, this interval can quickly become 5- or 6-hourly, given clinical judgement and excessive clinical workload [18, 21, 38–40]. Usually, the higher the target band, the greater the permissive hyperglycaemia, and, indirectly, the lower the risk of hypoglycaemia. However, it is also important to keep in mind it is impossible to clearly know whether the patient suffered from hypoglycaemia over longer measurement intervals without continuous glucose monitoring (CGM) or similar [41, 42].

**Fig. 5:**
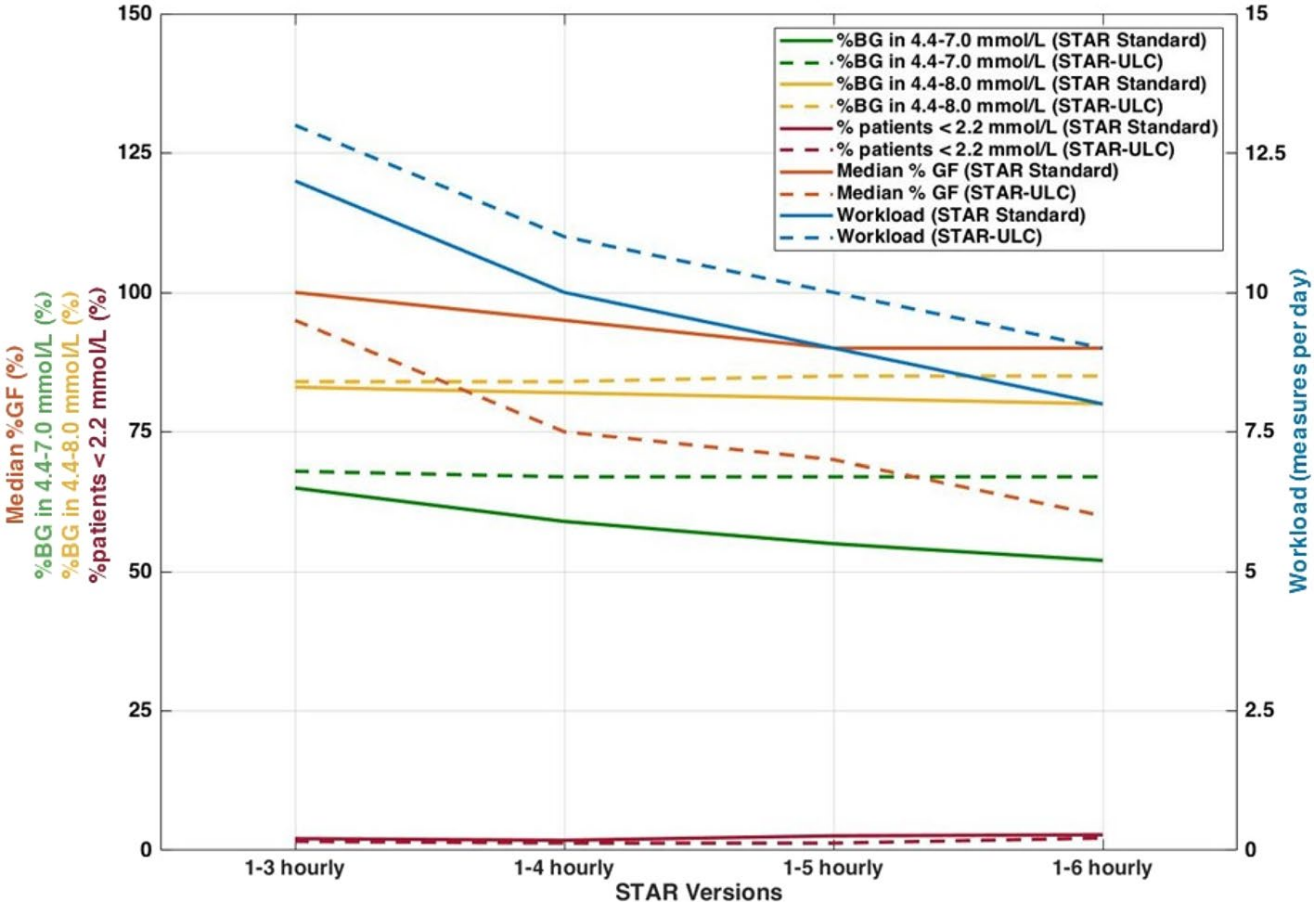
Risk and reward trade-off between STAR Standard (solid) and STAR-ULC (dashed) with increasing measurements intervals

This study assesses the potential to reduce workload with the safe, and effective STAR GC framework, and the impact on safety and performance. The results presented in this study clearly illustrate, and quantify for the first time, the risk and reward trade-off using longer measurement intervals in the context of STAR. Comparing STAR-3H and STAR-6H, the risks include higher incidence of severe hypoglycaemia (2.1% vs. 2.8%, respectively), lower %BG in intermediate bands (65% vs. 52% BG in 4.4–7.0 mmol/L, respectively, and 83% vs. 80% BG in target band), and lower nutrition rates achieved (100% vs. 90% GF, respectively), and the reward is the lower associated workload (12 vs 8 measures per day). When considering STAR-ULC to mitigate the associated increased hyperglycaemic risk with longer treatment intervals, improved performance is achieved compared to STAR-Standard, with similar safety, and this performance is consistent with the treatment intervals (68% vs. 67% BG in 4.4–7.0 mmol/L and 84% vs. 85% BG in target band for STAR-3H-ULC and STAR-6H-ULC, respectively). However, this gain in performance compared to STAR-Standard was achieved with relatively much lower median nutrition rates (95% and 60% GF for STAR-3H-ULC and STAR-6H-ULC vs. 100% and 90% for STAR-3H and STAR-6H), lower insulin rates (3.0 and 2.0 U/h for STAR-3H-ULC and STAR-6H-ULC vs. 3.2 and 2.0 U/h for STAR-3H and STAR-6H), and similar workload (13 and 9 measures per day for STAR-3H-ULC and STAR-6H-ULC vs. 12 and 8 measures per day for STAR-3H and STAR-6H). These results are summarised in Fig. 5.

Longer intervals are thus associated with increased risks in the context of STAR. Specifically, extreme changes in SI levels between consecutive measurements have a greater chance to occur as measurement intervals increase. Typically, for a given insulin dose and a sudden rise in SI, BG levels will suddenly drop. If this (unpredicted) drop occurs 1 h after treatment intervention and the next measurement is due in 5 h, it can have significant impact on patient BG, seen in the increased number of patients experiencing severe hypoglycaemia (Table 1). However, when limiting GC to lower measurement intervals, this sudden reduction in BG levels will potentially be seen sooner, and treatment adapted, potentially averting severe hypoglycaemia. This scenario is shown in Fig. 3, where Patient B becomes more insulin sensitive at 12 h, and where STAR-3H captures this behaviour and can adapt treatment faster (at 15 h) compared to STAR-6H where severe hypoglycaemia occurs (at 18 h).

Importantly, when STAR assesses the risks associated with a specific treatment for an interval longer than 1 h, the predicted SI range unique to each interval is used to predict the corresponding BG evolution range over the specific time period. The risks of extreme changes in SI for longer treatment intervals are thus considered for any treatment intervals. For example, when assessing the risks associated for a timeframe interval of 3 h, the evolution of BG is calculated based on the predicted SI_*n*+1_ for the first hour, SI_*n*+2_ for the second hour, and SI_*n*+3_ for the third hour. Thus, the extreme potential changes in SI levels between measurements, which are unique or different for each interval, are taken into account, as for each interval, the likely 5th–95th percentile prediction range of the evolution of SI is determined based on the current patient-specific identified metabolic state.

This outcome also emphasises the importance of accurately characterising intra-patient variability, where improved predictions would improve GC outcome. Ongoing studies are assessing the benefits of using more complex stochastic models [35, 43–45], and are currently being tested in clinical trials to validate the results. However, as this is a first study analysing longer treatment intervals, the well-proven original stochastic model approach is used here.

As seen in Figs. 2, 3 and 4, the different GC scenarios, based on the different measurement intervals allowed, led to significantly different measurement timing. Therefore, while one version could by chance measure BG right before hypoglycaemia, another could fail due to unfortunate timing based on prior treatment intervals selected. This issue adds difficulty when interpreting results, but reflects real practice, where measurement timing is also a factor influencing control. In clinical practice, despite nurse selection of a specific treatment interval, the new measurement may be taken a few minutes, or even hours, later/earlier. This measurement (mis)timing may thus (unexpectedly) influence results, as seen in Table 2, where incidence of severe hypoglycaemia is actually lower for STAR-ULC-4H (1.3% of patients) and STAR-ULC-5H (1.3% of patients) compared to STAR-ULC-3H (1.6% of patients). However, while this issue is typical in medical environment and time-dependent decision-making, a large cohort of virtual patients enables a balanced analysis of the potential advantage/disadvantage. More specifically, the differences reported in Table 2 are small, and may thus be considered, based on our experience, as simulation ‘noise’.

Another potential consequence of increased measurement interval is the lower confidence in future evolution of SI. The 5th–95th percentile of predicted SI is thus wider (Fig. 1), and STAR will consistently be more conservative in insulin dosing [43, 45], typically providing lower insulin rates to ensure safety (Table 1). While it is a safe approach, performance is affected due to the higher predicted risk, increasing BG levels (Table 1). The other effect is a general increase/shift in BG outcomes achieved, leading to higher %BG > 8.0 mmol/L and %BG > 10.0 mmol/L, the severe hyperglycaemic threshold. Interestingly, this outcome is achieved with slightly lower, but still high, nutrition rates [46, 47] to avoid potentially more important hyperglycaemic risk (Table 1).

Hence, to reduce the related expected increased hyperglycaemia, an adapted approach forcing the 95th percentile of predicted BG ≤ 8.5 mmol/L, the STAR-ULC approach, was undertaken. Figure 5 presents the main risk and reward summary outcome comparison between STAR Standard and STAR-ULC as a function of measurement intervals. Significantly more consistent GC outcomes were achieved regardless of measurement timeframe (Table 2, Fig. 5). Surprisingly, these results show improved safety, and lowered the number of patients experiencing hypoglycaemia. This result and consistency can be explained by the increased workload, increasing the chances to react faster to reduced BG. However, it is most likely due to treatments suggesting lower insulin and nutrition rates (Table 2, Fig. 5), where insulin’s impact on BG reduction from a sudden rise in SI was reduced thanks to lower insulin concentrations and concomitantly reduced nutrition.

Virtual patient trials using the STAR-ULC to mitigate the risk of hyperglycaemia due to larger predicted variability resulted in trade-off between BG outcomes, workload, and nutrition rates achieved. Nutrition management in ICU is a hot topic [48–51], where no clear uniform guidelines exist. Recent reviews suggest stepping increased nutrition rates from ICU admission, starting at 25% GF and ideally increasing by 25% every 2 days to reach 100% within a week [49]. In these results, nutrition rates achieved (60 [50 75] %GF in the worst STAR-ULC-6H case) are still comparable to, or better than, the recommendations, and thus potentially acceptable.

In addition, these rates achieved with STAR-ULC were comparable to the SPRINT protocol results, which was the only study to reduce all three of mortality, organ failure, and hypoglycaemia [36, 52]. Previous studies showed STAR using 1–3 h intervals provides close to the best nutrition delivery rates in the world [46] due to its ability to provide personalised nutrition, adapted to patient needs, while always ensuring safety. Hence, these results show the STAR Standard and STAR-ULC approaches can deliver acceptable, but different nutrition delivery rates with extended intervals and reduced workload, presenting a clear trade-off choice.

Ideally, 1-hourly measurements would provide the best outcomes. However, this approach is not clinically feasible and would require too much workload. CGM could also potentially provide improved control [42, 53]. In general, this technology is still not fully reliable in ICUs [54], but may develop further in future to full effect and enable far more flexible control approaches [55].

Overall, the virtual trial results are encouraging, and, regardless of measurement interval, provided safe and effective control for nearly all patients. Consistent high %BG in the tighter, safer 4.4–7.0 mmol/L and wider, still safe 4.4–8.0 mmol/L target band are associated with improved outcomes in ICUs [31, 36, 56]. Results suggest STAR is robust when using longer treatment intervals, and can safely adapt treatment to patient needs. However, these results also show the inevitable risk and reward trade-off between measurement interval and GC safety and efficacy. Increasing measurement intervals modestly increases risk of hypoglycaemia from 1.6% of patients to 2.2% or 2.8% (Tables 1, 2), which are still very low compared to many prior studies [57–61]. The potentially bigger trade-offs come between nutrition delivery and desired performance, both compared to workload.

More specifically, reducing workload using longer treatment intervals results in slightly high incidence of hyper- and hypo-glycaemia, given higher potential future SI variability. STAR-ULC provides safer, more effective, and tighter control compared to STAR Standard, at the cost of slightly increased workload and lower nutrition and insulin rates. This outcome suggests high nutrition and insulin rates magnify uncertainty as treatment interval increases, which should be expected. Reducing nutrition (and thus insulin) thus reduces risk of hypoglycaemia, further emphasising this “workload-performance-nutrition” risk and reward trade-off. While 4-hourly measurements are common in GC, whether 5- and 6-hourly are suitable in clinical practice is an important question.

The only major change in the STAR GC protocol design in this analysis is the ability to suggest longer treatment intervals, given these treatments meet safety requirements, using additional corresponding extended stochastic models. Nothing else was changed from the original protocol. However, further analysis could consider some kind of hybrid system, with more restriction for longer treatment intervals (such as a potential reduced upper limit of insulin rate), to avoid additional risks. While this change could be considered, results presented here still show very high safety compared to most published protocols [40, 57, 58], and, thus, such changes to the original protocol seem less necessary.

Comparison to other protocols is difficult as published studies often lack quality metrics and/or do not report results the same way [20, 62, 63]. Clinical protocols are effectively compared in the broad safety and performance metrics reported, but no specific table is given as there are so many reports of different lengths and intensity. Compared to major protocols, such as NICE-SUGAR, Glucontrol, and VISEP [5–7], the safety from hypoglycaemia at 1% by patients or less presented here is far better. Performance in time in band (estimated) is also much better, although median cohort BG is similar. Finally, workload for STAR-3H clinically and here [24] is higher, but for the STAR 4- to 6-hourly results, where nutrition modulated to limit the outcome BG range safety and performance, are far better than these well-reported studies, and workload is now similar and more clinically acceptable.

Compared to prior model-based analyses, not used clinically, such as the STOMP protocol, created by this group [64], the performance and safety are similar, but workload is far lower for the nutrition limiting versions. STOMP used longer interval stochastic models to minimise 3-hourly hypoglycaemic risk. It analysed each 3-hourly interval out to 6 h to ensure any dose given did not increase future risk due to either timing errors as clinical staff were busy or due to intervention choices leading to combinations that unknowingly made future treatment choices difficult or more risky. STOMP was never clinically implemented as the results were not a significant improvement on STAR as implemented, unlike those presented in the trade-offs here.

The results presented here use virtual patient and trial simulations [26]. Such simulations use a physiological model, where some physiological parameters are approximated, and, thus, could potentially lead to some minimal bias [65]. However, the model used has been validated and extensively clinically used in a wide range of clinical scenarios [24–27, 66–69]. It is also proven to reflect what is seen clinically by accurately predicting subsequent clinical results [23, 70]. However, virtual trials represent ideal conditions, with full compliance to protocol. Results may thus be a best case compared to reality, but representative of the reality and generalisable to other population cohort. Hence, all results presented should be validated in future clinical pilot trials, which are justified by the results presented here.

## Conclusions

In this study, the STAR GC framework is shown to provide safe, effective control to nearly all patients, despite increasing measurement intervals from 3- to 6-hourly to reduce workload. However, longer treatment intervals are associated with modestly increased risks of hyper- and hypo-glycaemia, as well as potential reductions in nutrition delivery when these risks are mitigated by limiting hyperglycaemic risk. The overall results present a clear risk and reward trade-off between workload and GC outcomes within the context of this proven risk-based GC framework. Overall, STAR’s unique risk-based dosing approach is robust to adaptation to using longer treatment intervals. Clinical pilot trials using STAR with different measurement timeframes should be undertaken to confirm these results clinically.

## Methods

### Patients and data

**Fig. 6:**
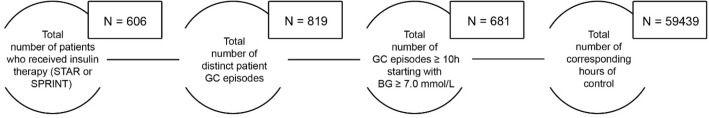
GC episode selection from the original 606 patients

Retrospective clinical data from 606 patients from different ICU settings are used. These patients underwent GC episodes using STAR (Christchurch, New Zealand and Gyula, Hungary) [24], and SPRINT [52], the ancestor of STAR (Christchurch, New Zealand). As patients can have multiple different GC episodes, this cohort includes 819 GC episodes, totalling 68,629 h of treatment. To avoid inconsistent and/or short GC episodes less representative of typical GC patients, only 681 episodes longer than 10 h and with starting BG > 7.0 mmol/L are used (Fig. 6). This cohort captures 59,439 h of control. Overall patient demographics are shown in Table 3. SPRINT and STAR in Christchurch were implemented as standard practice, and de-identified data audit and analysis were approved by the New Zealand Health and Disability Ethics Committee Upper South Regional Ethics Committee B (Ref: URB/07/15/EXP). STAR Gyula was also implemented as standard practice, and de-identified data audit and analysis is approved by the local ethical codes of Hungary.

**Table 3:** Summary of patient demographic data

	SPRINT Christchurch	STAR Christchurch	STAR Gyula
# Episodes	442	330	47
# Patients	292	267	47
# Hours	39,838	22,523	6268
% Male	62.7	65.5	61.7
Age (years)	63 [48, 73]	65 [55, 72]	66 [58, 71]
APACHE II	19.0 [15.0:24.5]	21.0 [16.0:25.0]	32.0 [28.0:36.0]
LOS-ICU (days)	6.2 [2.7, 13.0]	5.7 [2.5, 13.4]	14.0 [8.0, 20.5]

Data are given as median [IQR] where relevant

### STAR glycaemic control framework

**Fig. 7:**
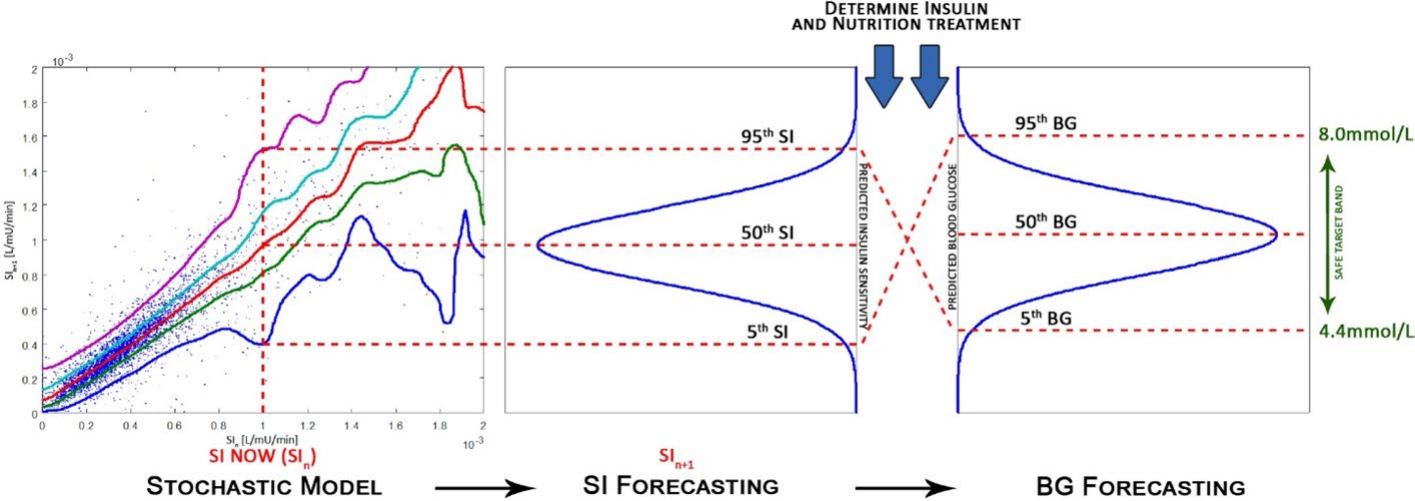
Risk-based dosing approach of the STAR framework. Current patient-specific identified SI is used to forecast the likely 5th–95th percentile range of future SI. This range is used to calculate the corresponding 5th–95th percentile range of likely future BG outcome for a given insulin and nutrition inputs

STAR is a model-based, patient-specific GC framework [23]. STAR uses a clinically validated physiological model along with a stochastic model to provide a unique risk-based dosing approach [34, 65]. Inter-patient variability is assessed by identifying model-based, patient-specific SI from patient data [71]. SI is a key physiological parameter characterising patient response to insulin [39]. Given current SI, the STAR stochastic model predicts a distribution of likely SI for 1–3 hourly intervals, directly quantifying the intra-patient variability of future SI evolution. It then uses the 5th–95th percentile range of future SI [34] to calculate the corresponding 5th–95th percentile range of predicted BG outcomes for a given insulin and nutrition input. STAR adjusts treatment choices to enable a pre-set, clinical risk of 5% of future BG below the clinically set target band lower limit of 4.4 mmol/L (or any pre-set clinical value), as shown in Fig. 7. The stochastic model in STAR is built on population data, using kernel-density methods [34].

Glycaemic and nutrition management are often considered independently [16, 33]. Most GC protocols thus only modulate insulin to reduce BG levels [22, 57–61]. However, to date, STAR is the only GC protocol also modulating nutrition to control glycaemia [23, 24, 33], where other may change nutrition levels in response to hyperglycaemia as recommended by nutrition guidelines [16], but not per protocol design. Nutrition is reduced if insulin alone is not sufficient to reduce excessive BG levels [23]. Typically, highly resistant patients quickly reach insulin saturation effect on BG uptake (6–8 U/h). For those patients, nutrition must (also) be reduced to lower BG to safe levels. Thus, nutrition can be temporarily reduced to a minimum of 30% original GF if insulin alone is not sufficient to safely control BG into the target band. Despite modulating nutrition, STAR has been shown to achieve close to best ICUs daily nutrition goals in the world, providing thus personalised nutrition [46].

STAR provides safe and effective control for nearly all (over 95% of) patients [24]. It typically targets the 4.4–8.0 mmol/L range, allowing up to 6-8U/h of insulin with a maximum 2 U/h increase from any prior intervention. Nutrition can be reduced to a minimum 30% GF, with a maximum decrease of 30% over successive treatments. Full details can be found elsewhere [23].

### STAR 1–6 hourly extension

STAR currently uses 1–3 hourly measurements to provide GC [23]. This interval was originally chosen based on Christchurch (New Zealand) ICU standards and conservative decisions to ensure high safety and efficacy [52, 72]. The average 11–12 measurements per day required can be an excessive clinical burden in other ICUs [28–30], which could lead to protocol non-compliance [37], potentially affecting GC outcomes [18]. Therefore, STAR is extended in clinically validated virtual trials to 1 to 4-, 5-, and 6-hourly treatment intervals, using extended 1 to 6-hourly stochastic models with the goal of assessing the safety and performance trade-offs at longer intervention intervals within this proven GC approach.

It is hypothesised there will be some loss of tighter control to narrower, potentially safer 4.4–7.0 mmol/L bands, but lesser loss of performance in the wider, but still safe 4.4–8.0 mmol/L band [31, 56, 73]. Major questions arise over safety from mild and severe hypoglycaemia [5, 74] over longer intervals, and any impact from any resulting reductions in nutrition delivery [50].

In this study, 1 to 3-, 4-, 5-, and 6-hourly versions of STAR are simulated to better capture the effect of increased measurement intervals on STAR GC safety and performance. These stochastic models are built from retrospective patient data, using kernel-density methods [34, 43, 45, 75], where SI is identified hourly from BG, insulin, and nutrition clinical data [71, 76]. SI pairs (SI_*n*_, SI_*n*+*i*_) for *i* = 1,2,…,6 are created and used to build each stochastic models.

Kernel-density methods enable to identify probability density function [77] of future SI_*n*+1_ knowing its current SI_*n*_ state, based on local data density. More specifically, SI can be considered as a first order Markov chain, where the conditional probability distribution of future SI_*n*+1_ depend upon its prior state SI_*n*_, which can be expressed:

$$P({\text{SI}}_{n + 1} |{\text{SI}}_{n} ,{\text{SI}}_{n - 1} , \ldots ,{\text{SI}}_{0} ) = P({\text{SI}}_{n + 1} |{\text{SI}}_{n} ) = \frac{{P({\text{SI}}_{n + 1} ,{\text{SI}}_{n} )}}{{P({\text{SI}}_{n} )}}.$$

**Fig. 8:**
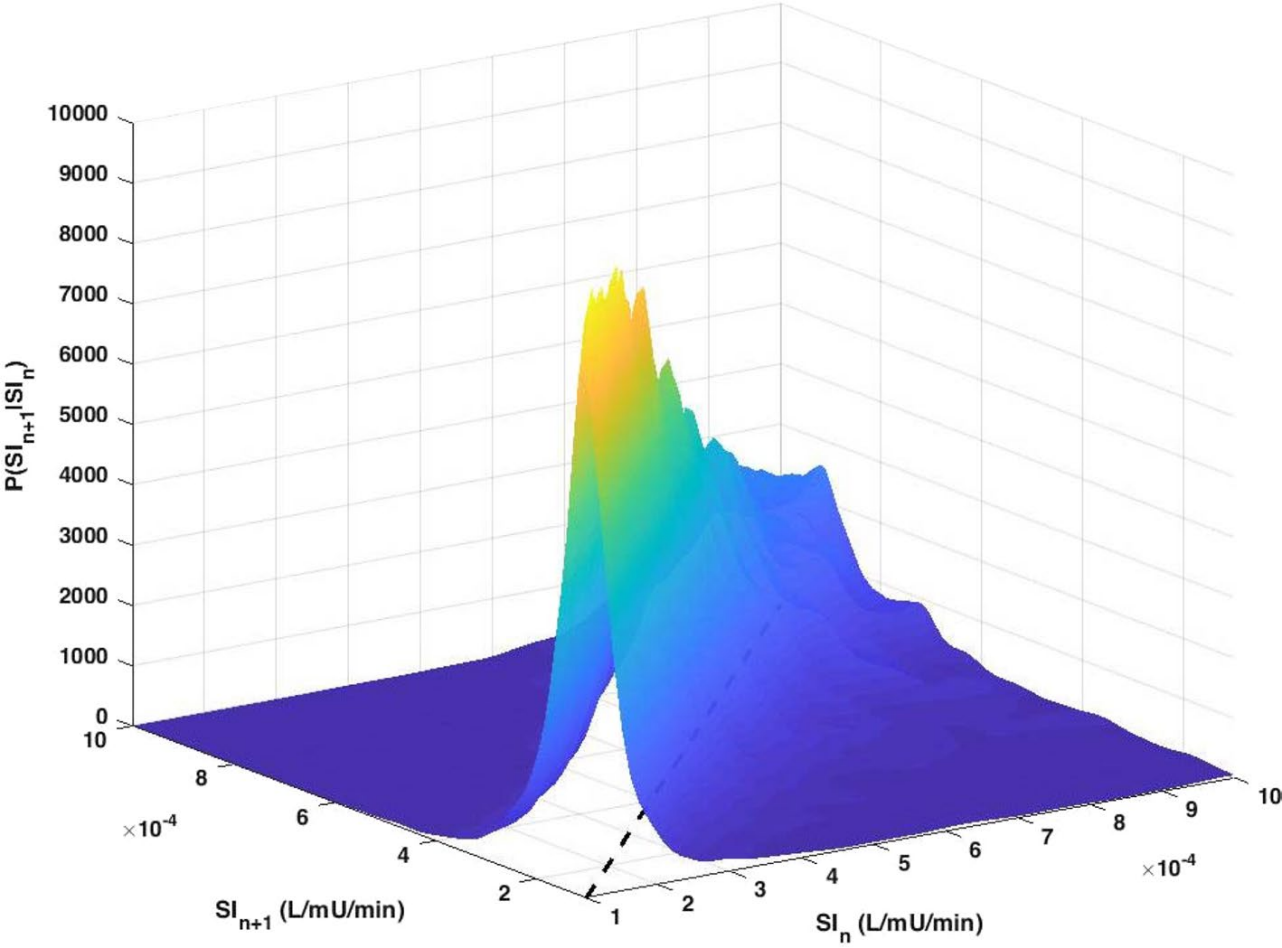
Stochastic modelling of SI_*n*+1_ variability. For each SI_*n*_ value, there exists a conditional probability distribution function (along SI_*n*+1_ axis) where the area under the curve sums up to 1.0

The joint probability $P({\text{SI}}_{n + 1} = y,\;{\text{SI}}_{n} = x)$ is determined using kernel-density methods and Gaussian estimator functions $\phi$, weighted according to local data density. More specifically, the 2D joint probability is the summation of these Gaussian distribution functions centred at each of the data points (*x*_*i*_,*y*_*i*_):

$$\begin{aligned} & P({\text{SI}}_{n + 1} = y,\;{\text{SI}}_{n} = x) = \frac{1}{n}\sum\limits_{i = 1}^{n} {\frac{{\phi (x;x_{i} ,\sigma_{{x_{i} }}^{2} )}}{{p_{{x_{i} }} }}} \frac{{\phi (y;y_{i} ,\sigma_{{y_{i} }}^{2} )}}{{p_{{y_{i} }} }}, \\ & p_{{x_{i} }} = \int_{0}^{\infty } {\phi (x;x_{i} ,\sigma_{{x_{i} }}^{2} )} , \\ & p_{{y_{i} }} = \int_{0}^{\infty } {\phi (y;y_{i} ,\sigma_{{y_{i} }}^{2} )} , \\ \end{aligned}$$

where $p_{{x_{i} }}$ and $p_{{y_{i} }}$ are used to normalise each Gaussian distribution function to the positive domain, ensuring a conditional probability such that $\int {P({\text{SI}}_{n + 1} = y|{\text{SI}}_{n} = x){\text{d}}x} = 1$ is satisfied for each SI_*n*_ values. More details on the methods used here are given in [34, 75]. Importantly, SI data are transformed into the logarithmic space to ensure the data to have a Gaussian distribution. An example of the resulting 1-h stochastic model is presented in Fig. 8.

Fivefold cross-validation is used to build new 1–6 hourly stochastic models using 80% of patient data (by patient). The resulting model is then tested using the new extended version of STAR on the other 20% of patient data, where all five test sets are reported in aggregated results. This approach ensures independent development and test sets and a more robust analysis ensuring stochastic models are not biased by outlying patients or small sub-cohorts.

While inter-patient variability is not equivalent between patients, intra-patient variability is equivalent regardless of patient conditions [19, 21, 26]. The different groups were thus randomly created from the original retrospective GC episodes, regardless of specific demographic characteristics. However, sufficient data density was ensured in each group to build the model, and sufficient GC episodes test the new model GC performance. Each group thus represents minimum 130 patients, totalling over 10,000 h of control. Finally, the results for any one group in the fivefold cross-validation were not notably different, thus indicating each group was comprehensive in the dynamics of the patient cohort, matching prior results showing as few as 3000–5000 h can capture a far larger cohort of dynamics [21, 34, 75].

### Virtual trials

To compare the impact of longer treatment intervals on GC outcomes, validated virtual trials are used to simulate different protocol designs on virtual patients [69]. Virtual trials are simulated on Matlab using a Java version of STAR. Virtual patients are characterised by their unique hourly identified SI profile, created from BG, insulin, and nutrition clinical data [27, 69]. This approach allows comparison of the safety and performance of the original STAR 1–3 hourly [23], with STAR 1 to 4-, 5-, or 6-hourly on the same underlying virtual patients [27]. In these in silico simulations, virtual patients, including starting BG levels and nutrition rates, were based on initial starting clinical data. Such trials reflect ideal conditions with full compliance to protocol, and have been validated and generalised to different ICU populations [26, 69].

Importantly, virtual trials automatically select the longest treatment intervals suggested and available. Thus, if only 1-hourly is suggested by STAR for safety reasons, virtual trials will select this treatment. However, if STAR assessment of risks results in allowing longer treatment intervals, then the longest available will be automatically selected. These trials are thus “blind” to any other potential factors, such as low BG levels, that could affect nurse treatment selection in clinical use. Additionally, the ICING model used [65] enables protocol simulation using exogenous insulin infusion, insulin boluses, or both, based on protocol design or ICU practices. In this study, insulin boluses are used in the simulations, as that is the standard of care in Christchurch, the main reference centre for this study.

### STAR Upper Limit Controlled

STAR, in its current version, always ensures safety and maximises efficacy, not allowing the 5th percentile of future BG below the lower limit of the target band (4.4 mmol/L), and choosing the insulin and nutrition intervention that best overlaps the target band, all of which is a function of the risk-based dosing approach. Because a 3-h measurement interval is relatively short in a clinical sense, the 95th percentile is rarely above 8.5 mmol/L, which is considered acceptable, and nutrition in this case is not decreased. There is thus no strict condition on the resulting 95th predicted percentile BG in the treatment decision-making, and a treatment can be considered by STAR despite potentially leading to mild hyperglycaemia. However, as measurement interval increases, wider 5th–95th percentile prediction range of BG is more likely to be larger induced by higher potential variability [35, 43–45], resulting in predicted 95th percentile BG potentially much higher than 8.5 mmol/L.

To mitigate this impact of rising hyperglycaemia over longer intervention intervals, a second version of the protocol is implemented. In this case, the 95th percentile of predicted BG must strictly be lower than 8.5 mmol/L for the treatment intervention to be considered, which is accomplished (where necessary) by further reducing nutrition and/or not offering longer treatment intervals as they do not strictly meet this condition. This approach will decrease the increased risk of hyperglycaemia and show improved efficacy, but could also increase workload and/or reduce nutrition delivery, both of which are clinically desirable “rewards”. This second protocol approach is denoted STAR-ULC (STAR Upper Limit Controlled).

The combination of analysing two STAR protocol approaches (STAR Standard and STAR-ULC) over extended 4–6 hourly intervals limits the analysis and provides the full range of possible performance and safety trade-offs.

### Comparison analysis

Most studies assessing GC outcome often lack quality metrics and/or do not report results the same way [20, 62, 63]. This study thus compares results of the proven STAR protocol [24] using commonly used and recommended metrics in the field [16, 56, 62, 63, 74, 78–84]. More specifically, safety, efficacy, BG achieved, insulin and nutrition rates, and workload are compared. BG is hourly resampled to allow fair comparison between protocols. Safety is compared using %BG outside target band (%BG < 4.4 mmol/L and %BG > 8.0 mmol/L) and %BG below severe hypoglycaemic threshold (%BG < 2.2 mmol/L). Performance is analysed using %BG in the 4.4–8.0 mmol/L target band and median BG levels achieved. Per-patient insulin (U/h) and nutrition rates (%GF) are also compared, and workload is assessed using average number of measurements per day.

Additionally, the proportion of patients with ≥ 50% BG in 4.4–7.0 mmol/L and 4.4–8.0 mmol/L are compared for each protocol. High percentage time in these bands, and low incidence of hypoglycaemia, are associated with improved outcomes in ICU patients [7, 12, 31, 36, 56, 73, 85, 86]. Hence, comparing the number of patients reducing/improving time in these bands provides a further outcome-based means to quantify whether patient GC outcomes improved, or not. The number of patients experiencing severe hypoglycaemia is also compared.

The main outcome of the study is to show and evaluate the risk and reward trade-off where:

- Risks are to the outcome resulting safety (hypoglycaemia), efficacy (performance of GC control), and nutrition provided,
- Reward is the lower workload, reflected by lower measurements per day with the longer treatment intervals used.

This study thus analyses STAR’s design robustness as measurement timeframes increases, where, as per protocol design, a reduction in workload (reward) is expected, but at the cost of reduced safety and performance (risks).

## Data Availability

The datasets used and/or analysed during the current study are available from the corresponding author on reasonable request. However, a subset of the data is publicly available in another journal: [87].

## Abbreviations

BG: Blood glucose
GC: Glycaemic control
ICU: Intensive care unit
IQR: Interquartile range
STAR: Stochastic targeted
STAR-ULC: STAR Upper Limit Controlled
SI: Insulin sensitivity
